# Damage Detection of Asphalt Concrete Using Piezo-Ultrasonic Wave Technology

**DOI:** 10.3390/ma12030443

**Published:** 2019-01-31

**Authors:** Wen-hao Pan, Xu-dong Sun, Li-mei Wu, Kai-kai Yang, Ning Tang

**Affiliations:** 1 School of Materials Science and Engineering, Northeast University, Shenyang 110819, China; pwh@sjzu.edu.cn (W.-h.P.); sxd@neu.edu.cn (X.-d.S.); 2 School of Materials Science and Engineering, Shenyang Jianzhu University, Shenyang 110168, China; lmwu@sjzu.edu.cn (L.-m.W.); yangkaikai@stu.sjzu.edu.cn (K.-k.Y.)

**Keywords:** asphalt concrete, damage detection, ultrasonic wave, velocity

## Abstract

Asphalt concrete has been widely used in road engineering as a surface material. Meanwhile, ultrasonic testing technology has also been developed rapidly. Aiming to evaluate the feasibility of the ultrasonic wave method, the present work reports a laboratory investigation on damage detection of asphalt concrete using piezo-ultrasonic wave technology. The gradation of AC-13 was selected and prepared based on the Marshall’s design. The ultrasonic wave velocities of samples were tested with different environmental conditions firstly. After that, the samples were destroyed into two types, one was drilled and the other was grooved. And the ultrasonic wave velocities of pretreated samples were tested again. Furthermore, the relationship between velocity and damaged process was evaluated based on three point bending test. The test results indicated that piezoelectric ultrasonic wave is a promising technology for damage detection of asphalt concrete with considerable benefits. The ultrasonic velocity decreases with the voidage increases. In a saturated water environment, the measured velocity of ultrasonic wave increased. In a dry environment (50 °C), the velocity the ultrasonic waves increased too. After two freeze-thaw cycles, the voidage increased and the ultrasonic velocity decreased gradually. After factitious damage, the wave must travel through or most likely around the damage, the ultrasonic velocity decreased. During the process of three point bending test, the ultrasonic velocity increased firstly and then decreased slowly until it entered into a steady phase. At last the velocity of ultrasonic wave decreased rapidly. In addition, the errors of the results under different test conditions need to be further studied.

## 1. Introduction

Asphalt concrete is a widely used material for roads and pavements owing to its advantages like seamlessness, easiness of repair, high driving comfortability and lower noise. In addition to exposure to harsh environmental elements during service, traffic loads, sunlight, rain and air result in premature failure of pavement by rutting, raveling and cracking. Hence, the inevitable aging of transportation infrastructure create significant security and economic risks. This raises a new concept, preventative maintenance [1,2,3,4]. It is an important way for extending the service life of pavement in a cost effective manner. It change the passiveness to positive at the appropriate time.

For pavement maintenance, how to identify the damage and fatigue of pavement and make a cost-effective decision is a difficult task for engineers, especially appropriate time and frequency of maintenance. In the traditional process of evaluating and inspecting, the core samples are always drilled and taken to the laboratory to obtain the corresponding performance through standard test, so that the identify process is complicated and the test period is too long [5,6,7,8]. Furthermore, traditional maintenance is a passive way to repair the pavement when the pavement structure damage. It is very important to find a way to identify the maintenance time accurately and quickly. Therefore, some new non-destructive testing (NDT) techniques are applied in pavement assessment, such as ground-penetrating radar (GPR), spectral analysis of surface wave (SASW) and ultrasonic testing technology (UT) [9,10,11,12,13]. 

GPR is an electromagnetic wave method for detecting the internal structure of pavement by using high frequency pulse electromagnetic field, which can provide high resolution 2D and 3D under pavement images [14,15]. SASW has been widely used as a non-destructive testing method for underground exploration and geological experiment. The dispersion characteristics of the propagating surface wave in the pavement are measured by the phase difference between the two sensing channels. The dispersion curve is obtained by data transmission. The shape of the pavement and the shear velocity are estimated by the inversion program [16,17]. However, these two methods need to be tested in the field and analyzed in laboratory. Hence, how to realize the complete, fast and automatic recognition of the pavement damage in the field and how to guarantee the high accuracy rate, are the research topic that the relevant scholars need to solve urgently at present.

Ultrasonic testing technology is one of the most widely used nondestructive testing methods. The principle of ultrasonic testing technology is to excite elastic waves in engineering structures or materials through ultrasonic transducers. The elastic waves propagate in materials or structures with various waveforms and another transducer receives them. The characteristic parameters of elastic wave are directly related to the properties of materials or structure such as time, wave velocity, wave amplitude and wave shape. 

The application of ultrasonic testing technology is in the rock and cement concrete materials [18,19] but the application in asphalt mixture is not as many [20,21,22,23,24,25]. The research is still in the exploratory stage, such as the relationship between ultrasonic velocity and airvoid, elastic modulus, fatigue life and so on. Research still needs to supplement more data to improve the research system, so as to achieve qualitative to quantitative transformation.

In this study, the gradation of AC-13 was selected and prepared based on the Marshall’s design. The samples included Marshall specimens and beams. The ultrasonic wave velocities of samples were tested with different environmental conditions firstly like wet, dry and freeze-thaw. After that, the samples were destroyed into two types, one was drilled and the other was grooved. The ultrasonic wave velocities of pretreated samples were tested again. Furthermore, the relationship between velocity and damaged process was evaluated based on three point bending test.

## 2. Materials and Methods 

### 2.1. Materials

Bitumen, AH-90 paving grade, was obtained from Liaohe Oilfield of Panjin City, China. The physical properties of the bitumen are given in Table 1. 

### 2.2. Preparation of Asphalt Concrete 

Gradation of AC-13 was selected and 24 samples were prepared for optimal binder percentage according to Marshall’s design. The specimens were cylinders with a diameter of 101.6 mm and a height of 63.5 mm. The passing rate of AC-13 gradation for each sieve is given in Table 2. Depending on the results optimal binder percentage was 4.9%.

### 2.3. Experimental Methods

● Piezo-ultrasonic velocity test

The ultrasonic velocity test was carried out on TICO tester (Tectus Group, Zurich, Swiss). The testing voltage of ultrasonic wave was 250 V, the testing temperature was 15 °C and the frequency of ultrasonic wave was 30 kHz. Five test areas were selected for each specimen surface and the test results were averaged. In order to avoid the discreteness of collected data, a minimum of ten repeated measurements under the same experimental conditions (statistical sample size) would be necessary. Furthermore, five samples were prepared for each test.

● Computed Tomography

The scan images of asphalt concrete for voidage calculation were analyzed by an X-ray 3D microscope (nanoVoxel-2700, Sanying Precision Instruments Co. Ltd., Tianjin, China).

● Factitious damage

**Figure 1:**
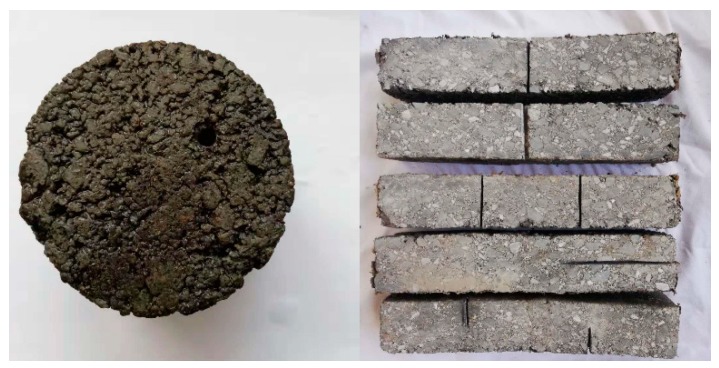
The factitious damage of specimens.

There were two types of factitious damage as shown in Figure 1. One is that a cylindrical through-hole of 5 mm diameter was drilled in the Marshall specimen. 

The other is that some grooves with different width were carved on the surface of asphalt beam but the depth of grooves was same, 15 mm. So there are five types of asphalt beam with different grooves as following: groove of 2 mm (G-2 mm), groove of 4 mm (G-4 mm), two grooves of 2 mm (DG-2 mm), vertical groove of 2 mm (VG-2 mm) and half double groove of 2mm (HG-2 mm).

● Drying and water-saturated test

The drying test is that all the samples were placed in an oven at 50 °C for 24 h. The water-saturated test is that all the samples were immersed in water for 24 h. The ultrasonic velocity of samples were measured directly after the environmental pretreatment.

● Freeze-thaw cycle

There were two cycles. All the samples were full immersed in water for half an hour first and then kept vacuum saturation state for 15 min. After pretreatment, the samples were frozen in the refrigerator at −18 °C for 16 h and then took them out and placed in a bath box at 60 °C for melting 6 h [26]. 

● Three point bending test

The three point bending test was carried out on a material testing machine (WDW-T200, Tianchen, Jinan, China). The distance between the two fulcrums was 200 mm and the loading point was located at the center of the specimen in length direction. The test was carried out at a dead loading rate of 0.2 kN/s. At the same time, the ultrasonic wave detector was applied to monitor the travel velocity in the specimen in real time until the specimen was damaged.

● Healing

After heating, the bitumen was coated on the cross section of the asphalt beam and then the two cross section were quickly bonded together. At last the healed asphalt beams were placed in an oven at 50 °C for one hour.

## 3. Results and Discussion

### 3.1. Ultrasonic Test

**Figure 2:**
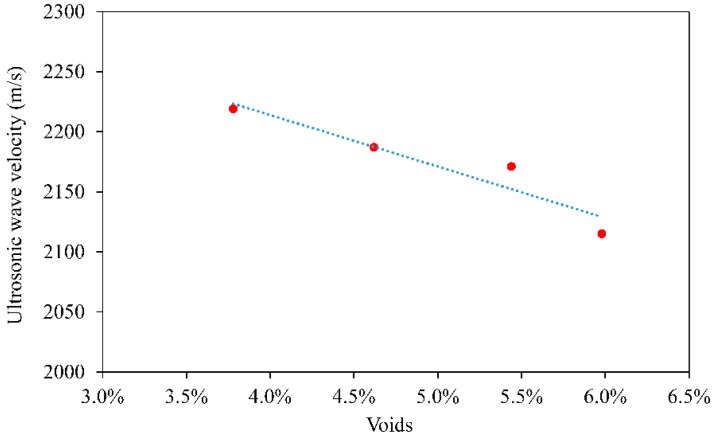
Relationship between ultrasonic wave velocity and voidage of the Marshall samples.

As a key factor, air voids has been paid much attention due to it is closely related to the road performance. The pavement with larger voidage has better structure depth, anti-skid performance and friction coefficient but the performance of anti-seepage, anti-freezing and anti-moisture is poor. Therefore, to establish the relationship between voidage and piezoelectric ultrasonic velocity is the first prerequisite for using piezoelectric ultrasonic technology. Based on the same gradation, different compaction temperature is used to control the voidage of asphalt concrete. The relationship between voidage and ultrasonic velocity are shown in Figure 2.

In Figure 2, the data points and trend line show the relationship between the ultrasonic wave velocity and the voidage of the Marshall samples. It is well known that the lower the compaction temperature, the higher the voidage. The voidage was 3.78%, 4.62%, 5.44% and 5.98%, respectively. Furthermore, the ultrasonic velocity decreased with the voidage increased. Obviously, there are a lot of voids in the asphalt mixture and the wave travels much faster in the air than in the aggregate, the higher the porosity, the smaller the velocity. In addition, the dispersion of the longitudinal wave was larger due to the high voidage, resulting in long travel time of ultrasonic wave and low wave velocity.

**Figure 3:**
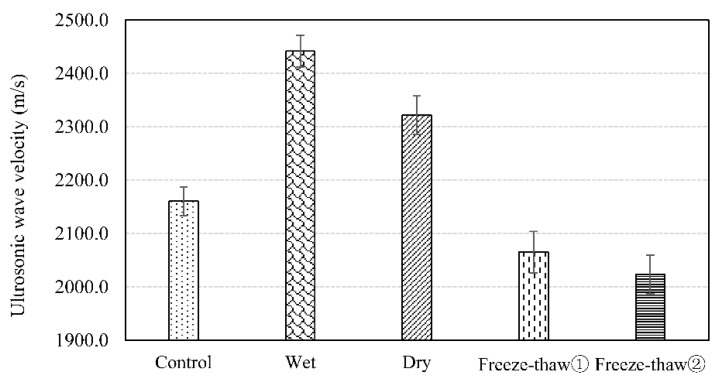
Ultrasonic velocity of Marshall specimens with different environmental conditions.

Usually the voidage of AC-13 asphalt concrete is controlled at 4–6%. In the following tests, the voidage of all Marshall specimens were controlled at 4.5% by compaction temperature. The ultrasonic velocities of Marshall specimens with different environmental conditions show in Figure 3.

In a saturated water environment, the average velocity of specimens was 2442 m/s. The reason is that the water in the pores increases and the pores are basically filled with water. In addition, the velocity of ultrasonic wave propagation in water was much faster than that in air. The increase of water caused the measured wave velocity to become bigger.

In a dry environment (50 °C), the average velocity of specimens was 2321 m/s. This data was higher than that of the control group and lower than that of the saturated environment. For bitumen, the higher the temperature is, the more obvious the viscoelasticity is. In addition, the dry temperature is higher than the softening point of bitumen. The ultrasonic waves face a greater viscous resistance and the interference and resistance to the propagation of ultrasonic waves are bigger. 

Furthermore, the velocity decreased in turn after two freeze-thaw cycles. Bitumen shows viscoelasticity at high temperature and elasticity at low temperature and occurrence of aging in the repeated process of high temperature and low temperature. In addition, the voids of asphalt concrete is occupied by water in the saturation environment. When water freezes, it becomes larger in volume instead of smaller and there is not enough void volume to accommodate the increased volume, so the expansion stress is generated in the void, so that the voidage increased and the ultrasonic velocity decreased gradually and the asphalt mixture was damaged at last.

### 3.2. Damage Detection

**Figure 4:**
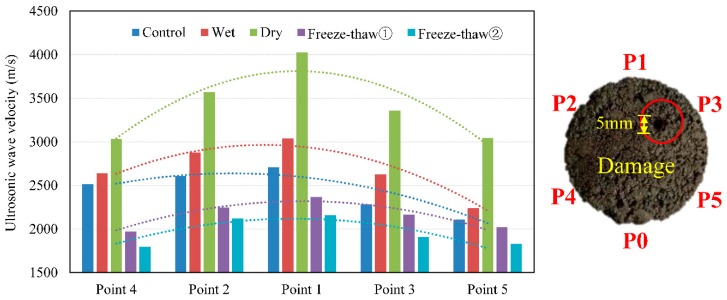
Ultrasonic velocity with different environmental conditions after factitious damage.

All the tests were repeated after the Marshall specimens had been drilled. The ultrasonic data collection included five receiver positions (P1 to P5) for one generator position (P0). Obviously, the distance at which the ultrasound wave travels was different and symmetrical, except P3, due to the damage. The test results show in Figure 4.

Figure 4 shows typical traces for an ultrasonic velocity at different receiver positions. For different environment, the collected ultrasonic data at each receiver position were similar to the data before the samples were damaged. The velocity increases under saturated and dry conditions and decreases after freeze-thaw cycles. In addition, with the increase of the wave travel length, the velocity of the ultrasonic wave in the dry environment increases rapidly, the maximum data is 4027 m/s. 

For different receiver, the wave moved from generator P0 to receiver P4, P2 and P1, the velocity increased (the travel lengths also increase). At receiver P1, the velocity of all samples further increased. On the symmetrical side with the damage, similarly the plot of the average measured velocities showed a dip in the measured velocity caused by the presence of damage. Note that as the velocity of receiver P3 was slower than that of P2 and the trends of average velocity were expected. This is clearly because the wave must travel through or most likely around the damage.

**Figure 5:**
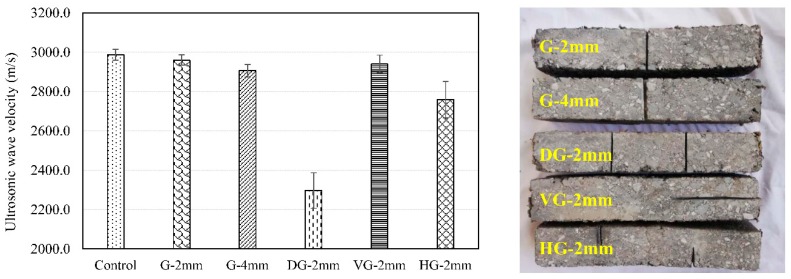
The velocity of ultrasonic waves with five types of grooves.

But the contact between side surface of the cylinder specimen and the sensor probe is not a complete contact but a linear contact. It can result in minor inaccuracies. To further study the influence of cracks on the velocity of ultrasonic waves, four types of grooves are carved on the different asphalt beam specimens. Figure 5 shows the test results of velocity with simulated cracks.

In Figure 5, for cracks width, ultrasonic velocity slightly decreased when 1mm grooves appear on the surface of asphalt concrete beam specimens. But when the crack width increases to 2 mm, the ultrasonic velocity decrease more. Note that the effect of fine cracks on ultrasonic velocity is slight and the ultrasonic velocity decreased with the increased of crack width. 

Furthermore, with the presence of two grooves, the ultrasonic velocity decreased obviously and the deviation of the test results was 90.74 m/s, which was very large. When an ultrasonic wave passed through a crack, only part energy was transmitted and the air inside the crack attenuated the ultrasonic wave. When the ultrasonic wave passed through the next crack, the velocity decreased even more. In addition, the anisotropic property of asphalt concrete makes the instability of the results. Because even if the grooves were in the same position, the aggregate and bitumen content of different bitumen concrete beams were also different at the grooves. 

Moreover, the two grooves have been halved. The test produced the velocity of ultrasonic wave ranging from one groove to two grooves. But the deviation of results was 93.21 m/s, which was very large, too. It revealed that there are significant positive correlations among simulated crack length, simulated crack position and ultrasonic velocity.

At last, asphalt pavement damage has not only transverse crack but also longitudinal crack. One groove was carve on the surface of asphalt beam as simulated vertical crack. The result shows the ultrasonic velocity decreased slightly. Hence, when the travel direction of ultrasonic wave was parallel to the crack direction, the change of ultrasonic velocity was slight.

### 3.3. Three Point Bending Test

**Figure 6:**
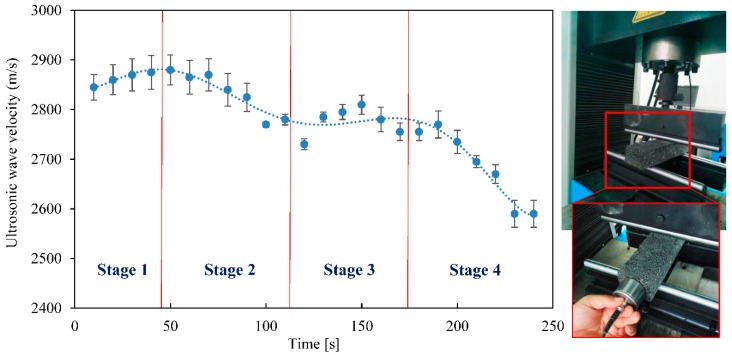
The change trend of ultrasonic velocity during the whole process of damage.

Three-point bending test is one of the methods to evaluate the fatigue and damage of asphalt concrete. The velocity of ultrasonic wave was measured when asphalt beam specimens were applied a load until failure. Figure 6 shows the change trend of ultrasonic velocity during the whole process of damage. In Figure 6, according to the slope of curve, the damage process of asphalt beam can essentially be broken down into four phases: loading, crack growth, balance and damage.

In the stage 1, the ultrasonic velocity increased firstly. In the first 50 s, the ultrasonic velocity increased slightly, because the flexural-tensile stress was applied to the asphalt beam, the travel lengths of ultrasonic wave increased. In addition, asphalt mixture is a typical heterogeneous material. Although the interface conditions between aggregate and bitumen is good, the mechanical properties of these two materials are different, so the deformation of asphalt mixture is very complex. Moreover the voids in the asphalt mixture occurred about 4.5% of the volume and these voids were crowded during the loading process.

In the stage 2, with the time increase (50 s to 100 s), the velocity of ultrasonic wave decreased slowly due to presence of some micro-cracks in the asphalt concrete beam. The growth rate of micro-cracks was higher than the compacted rate of the voids in asphalt concrete beam. 

In the stage 3, after 100 s, the velocity of the ultrasound wave barely changed and it had entered into a steady phase. This is clearly because the displacement of the aggregate and bitumen is caused by the deformation of asphalt beam at the same time. And the growth rate of micro-cracks was close to the compacted rate of the voids.

In the stage 4, the velocity of ultrasonic wave decreased rapidly after 170 s. The micro-crack width increased visibly and cracks appeared on both sides of the larger aggregate at the lower edge of the asphalt beam. The crack grew to the critical state of penetrating and then the asphalt beam was damaged.

In addition, the velocity of ultrasonic wave in the width travel direction of the asphalt beam was measured before and after the three-point bending test. Moreover, the damaged asphalt beam was coated with new bitumen and heated up for healing. All the results were showed in Figure 6.

**Figure 7:**
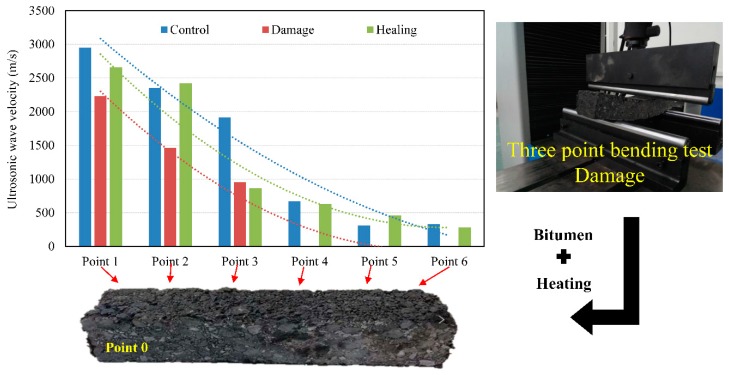
The velocity of ultrasonic wave in the width travel direction of the asphalt beam.

In Figure 7, with the increase of received length, the velocity of ultrasonic wave decreased one by one in any case. In addition, the damaged asphalt beam cannot receive ultrasonic wave at the receiving point P4 but the ultrasonic wave can be detected again after healing. It indicates that ultrasonic testing technology can be used to detect the cracking state of asphalt concrete effectively.

### 3.4. Impact Analysis

**Figure 8:**
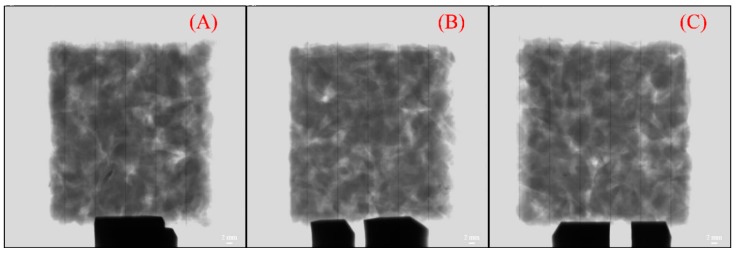
The CT images of cube samples with different voidage: (**A**) 4.08%, (**B**) 4.32%, (**C**) 4.56%.

Asphalt mixture is a typical heterogeneous material, so different measured positions, different results. In order to analyze the errors, the Marshall specimens were cut into a cube with a length of 5 cm on the side. This size is close to the size of the piezoelectric ultrasonic probe. The CT scan images are shown in Figure 8. Through the calculation of image processing software (nanoVoxel-2700 system), the voidage of three specimens is 4.08% (A), 4.32% (B) and 4.56% (C), respectively.

The ultrasonic velocity of three specimens was 2275 m/s, 2221 m/s and 2189 m/s, respectively. The relationship between velocity and voidage remains unchanged but the value was lower than that of the Marshall specimen. Hence, voidage, temperature sensitivity, viscoelasticity, travel length of ultrasonic wave in asphalt concrete and the size of specimens are main factors affecting the ultrasonic velocity.

Compared with other studies, the ultrasonic velocity is lower than 3000 m/s in ambient [21,22,23,24,25]. Based on the impact analysis, it reveals that the ultrasonic velocity is the result of the combined effect of temperature, environment condition and service time. Furthermore, it is still contingency because of the reflection, attenuation, resonance and changes of velocity that occurred in an object when the propagation of ultrasonic wave. Hence the research requires a variety of supplementary data. The results of this paper are a useful supplement.

## 4. Conclusions

This study has indicated that piezoelectric ultrasonic wave is a promising technology for damage detection of asphalt concrete with considerable benefits. The test results demonstrated that the ultrasonic velocity decreased with the voidage increases. Furthermore, the increase of humidity and temperature lead to an increase of ultrasonic velocity. Oppositely, after two freeze-thaw cycles, the voidage increased and the ultrasonic velocity decreased gradually.

For factitious cylinder damage, the velocity of ultrasonic wave decreased at damaged position. For factitious groove damage, ultrasonic velocity decreased slightly with the increased of crack width but decreased obviously with the increased of crack quantity. In addition, the change of ultrasonic velocity was slight when the travel direction of ultrasonic wave was parallel to the crack direction.

For the relationship between ultrasonic velocity and the damaged process of asphalt beam, the damaged process can essentially be broken down into four phases: loading, crack growth, balance and damage. Correspondingly, the ultrasonic velocity increased firstly and then decreased slowly until it entered into a steady phase. At last the velocity of ultrasonic wave decreased rapidly.

In addition, voidage, temperature sensitivity, viscoelasticity, travel length of ultrasonic wave in asphalt concrete and the size of specimens are main factors. The errors of the results under different test conditions need to be further studied.

## Figures and Tables

**Table 1:** Properties of bitumen.

Property	Unit	Results	Technical Requirements	Test [26]
Penetration	dmm	88.2	80–100	T0604
Ductility	cm	127	≥100	T0605
Softening point	°C	49.1	≥45	T0606
Density	g/cm^3^	1.03	–	T0603

**Table 2:** Aggregate gradation for AC 13.

**Sieve Size (mm)**	16	13	9.5	4.75	2.36	1.18	0.6	0.3	0.15	0.075
**Passing (%)**	100	94.8	77.1	48.6	30.3	22.8	16.2	11.4	7.6	6.1
